# Numerical Investigation of Conventional and Ultrasound-Assisted Aqueous Extraction of Caffeine from Whole Green Robusta Coffee Beans: Extraction Enhancement via Changing of Extraction Water

**DOI:** 10.3390/foods14111956

**Published:** 2025-05-30

**Authors:** Kuson Bawornruttanaboonya, Nathamol Chindapan, Sakamon Devahastin

**Affiliations:** 1 Advanced Food Processing Research Laboratory, Department of Food Engineering, Faculty of Engineering, King Mongkut’s University of Technology Thonburi, 126 Pracha Uthit Road, Thung Khru, Bangkok 10140, Thailand; 2 Department of Food Technology, Faculty of Science, Siam University, 38 Phetkasem Road, Phasicharoen, Bangkok 10160, Thailand; 3 The Academy of Science, The Royal Society of Thailand, Lak Si, Bangkok 10210, Thailand

**Keywords:** decaffeinated coffee, mass transfer, mathematical modeling, moisture content, process intensification, sonication

## Abstract

To enhance the low-efficiency but potentially health and environmentally friendly aqueous decaffeination process, ultrasound-assisted aqueous extraction (UAAE) has recently been proposed. A novel concept of intermittent extraction water change to further enhance UAAE has also been mentioned, but not yet studied in detail. For this reason, a mathematical model that can be used to predict the concentration evolutions of caffeine during UAAE and conventional aqueous extraction (CAE) of whole green robusta coffee beans is herein proposed. The model consists of terms representing transient intra-bean caffeine and water diffusion as well as molar fluxes of caffeine and water on the bean surface. After validation, the model was used to investigate the effects of extraction temperature, bean-to-water mass ratio and frequency of extraction water change on caffeine concentration evolutions. Simulation results show that UAAE exhibits around 10% higher caffeine removal rates than CAE at all investigated conditions. Extraction temperature of 70 °C, bean-to-water ratio of 1:3, and extraction water change at every 1 h interval are noted as the most appropriate conditions for UAAE. The required extraction durations of UAAE under these conditions are 13 h and 24 h to meet the US and European Union standards, respectively.

## 1. Introduction

Caffeine (1,3,7-trimethylpurine-2,6-dione) is a dominant compound in coffee and one of the compounds responsible for the popularity of the beverage. Caffeine stimulates the central nervous system and therefore helps increase energy and decrease fatigue [1]. Caffeine has also been reported to enhance athletic performance, promote weight management, and protect against several chronic diseases, such as dementia and liver and heart diseases [1,2,3]. Caffeine intake lower than 400 mg/day is noted to be safe for adults [4,5]. Excessive daily intake of caffeine has, on the other hand, been reported to possibly lead to several adverse side effects, including anxiety, insomnia, digestive issues, and even muscle breakdown [6,7,8,9]. Excessive consumption of caffeine during pregnancy may increase the risk of miscarriage or result in low birth weight [10,11].

To respond to the demand of some consumers who prefer caffeine-free coffee, extraction of caffeine from green coffee beans prior to roasting has long been conducted. Caffeine can be extracted by several methods, e.g., organic solvent (e.g., dichloromethane and ethyl acetate) extraction, supercritical carbon dioxide (CO_2_) extraction, and aqueous extraction. Coffee beans are nevertheless noted to suffer losses of aroma and flavor compounds after organic solvent extraction [12]. Residual organic solvent in a decaffeinated product may also pose a concern [13]. While supercritical CO_2_ extraction can alleviate the aforementioned limitations and disadvantages, this extraction technique is very costly [12,14].

As caffeine is water-soluble, aqueous extraction is an attractive alternative for decaffeination. The best-known aqueous extraction process is the patented Swiss Water process [15], which uses only water to remove up to 99.9% of caffeine in green coffee beans. Aqueous extraction nevertheless exhibits lower efficiency when compared with other extraction methods. The required extraction time is very lengthy [12].

To enhance decaffeination efficiency, ultrasound-assisted extraction has been proposed and tested. Ahmad et al. [16], for example, studied the removal of caffeine from green robusta coffee powder by using natural deep eutectic solvent composed of betaine and sorbitol with the assistance of ultrasound. Such ultrasound-assisted extraction was noted to be as much as fourfold more efficient than conventional reflux extraction using 70% ethanol as the solvent. However, comparison of caffeine removal with and without the assistance of ultrasound was not conducted. It is therefore not certain whether the improved removal efficiency was due to the use of ultrasound or simply to solvent replacement. Other studies that confirm the capability of ultrasound to enhance the efficiency of caffeine removal are available. Tang et al. [17], for instance, conducted ultrasound-assisted supercritical CO_2_ extraction of caffeine from green tea powder at a temperature of 60 °C and a pressure of 25 MPa. These investigators noted that the amount of caffeine that could be removed with the assistance of ultrasound was higher than that without the use of ultrasound by about 30%. No prior study is, however, available on ultrasound-assisted aqueous extraction of caffeine, especially from whole green coffee beans. Only coffee bean powder was used as raw material in the past. Note that the transport behavior within a whole coffee bean and that in coffee powder is expected to be much different. The difference is in turn expected to significantly affect the process of extraction.

To further enhance UAAE, a novel concept of intermittent extraction water change has been mentioned, but not yet studied in detail [18]. Extraction water change at time intervals is hypothesized to help break the equilibrium between the caffeine on the bean surface and that in the extraction water. The caffeine concentration gradient, which drives the extraction process, could therefore remain at a higher value throughout the extraction process. However, it is not known how often extraction water change should be performed. A large number of experiments would need to be conducted to arrive at an appropriate water change frequency. This is a tedious and time- and resource consuming task. An alternative means to an exhaustive experimental program is needed.

Based on the abovementioned arguments, a mathematical model that can be used to predict the concentration evolution of caffeine during UAAE and the more conventional aqueous extraction (CAE) of whole green robusta coffee bean is proposed. Robusta coffee was chosen as it is known to contain a higher amount of caffeine than its arabica counterpart [19]. Selected experiments were conducted to validate the proposed model. The validated model was then used to numerically study the effects of the extraction water temperature, bean-to-water ratio, and frequency of extraction water change on the evolution of caffeine concentration within a coffee bean. A condition providing maximum caffeine removal capability was then identified.

## 2. Materials and Methods

### 2.1. Materials

Whole green robusta coffee beans with an initial moisture content of 12%–14% (d.b.) were purchased from Chumphon Coffee Planters Cooperative Group (Tha Sae district, Chumphon province, Thailand). The beans were packed in a polyethylene bag, which was then placed in a sealed plastic container at room temperature until further use.

Pharmaceutical-grade magnesium oxide was obtained from AppliChem GmbH (Darmstadt, Germany). Analytical-grade dichloromethane was obtained from Loba Chemie Pvt. Ltd. (Mumbai, India). Standard-grade caffeine was obtained from Sigma-Aldrich (Shanghai, China).

### 2.2. Experimental Methods

#### 2.2.1. Preparation of Green Coffee Beans

Around 120 g of green coffee beans was soaked in water. The mass ratio of the beans to water was around 1:2 to fully cover the beans. Floated (defected) beans were removed from the water, while the rest were left for 10 min. After draining, moisture content of the beans was in the range of 20%–22% (d.b.). Soaking was conducted to soften the bean structure, hence making the beans ready for the subsequent extraction process.

#### 2.2.2. Conventional and Ultrasound-Assisted Aqueous Extraction

In the case of conventional aqueous extraction (CAE), the prepared green coffee beans (100 g) were mixed with distilled water at a bean-to-water mass ratio of 1:3 in a 500 mL Erlenmeyer flask. The flask was closed with a stopper and placed in a water bath (Memmert, WNB 14, Schwabach, Germany). The mass ratio of 1:3 was selected based on the results of our preliminary experiments, which indicated that such a ratio could help accelerate caffeine removal while resulting in the least extensive deterioration of the bean structure. The extraction process was conducted at 70 °C for either 2, 4, 6, 8, 10, 12, or 14 h. In cases where the extraction water was to be changed and where the extraction time was longer than 2 h, the water was changed every 2 h. It is important to note that these selected conditions were not yet optimized. Each batch of extracted beans was dried at a lower temperature of 40 °C for 18 h to avoid changes in the bean structure due to thermal degradation prior to the analysis of the caffeine content.

In the case of ultrasound-assisted aqueous extraction (UAAE), the bean preparation procedures were the same as those in the case of CAE. However, an ultrasonic bath (Witeg, WUC-A0317, Wertheim, Germany), operated at a frequency of 40 kHz and input power of 75 W, was used instead of the water bath. The extraction process was again conducted at 70 °C for 2, 4, 6, 8, 10, 12, or 14 h, either with or without changing of water every 2 h. Each batch of extracted beans was also dried at 40 °C for 18 h prior to the analysis of the caffeine content.

#### 2.2.3. Determination of Bean Moisture Content

Moisture content of coffee beans (5 ± 1 g) was determined by the standard gravimetric method [20] at 105 °C for 72 h in a hot-air oven (Memmert, UF 260, Schwabach, Germany).

#### 2.2.4. Determination of Bean Dimensions and Volume

Dimensions of coffee beans were measured in terms of height, width, and thickness using a Vernier caliper (Tricle, Shanghai, China; accuracy of ±0.05 mm). Fifty beans were measured, and the average dimension values were used for the simulation.

In the present study, the shape of a green coffee bean was assumed to be semi-ellipsoidal. The volume of a bean can then be calculated using Equation (1):

(1) $$V_{bean}=\frac{1}{2}\times \frac{4}{3}\pi (abc)$$

where $V_{bean}$ is the calculated volume of a green coffee bean (m^3^), while *a*, *b*, and *c* are the height, width, and thickness of the bean, respectively [21].

#### 2.2.5. Determination of Bean Caffeine Content

Caffeine was first extracted from the beans as per the methods of Chindapan et al. [22]. The beans were ground in a grinder (Calino, 600N, Taiping, Taiwan) and sieved through a 60-mesh screen (Retsch, Haan, Germany). Around 1 g of the sample was weighed into a 400 mL Erlenmeyer flask, and 5 g of magnesium oxide and 250 mL of distilled water were added. The mixture was heated at 90 °C and stirred for 20 min. After cooling to room temperature, the sample was filtered through Whatman no. 4 filter paper. The filtered sample was diluted with distilled water to fill a 250 mL volumetric flask.

Determination of the caffeine content in the prepared sample was conducted as per the methods of Belay et al. [23]. Briefly, 25 mL of the sample extract was mixed with 25 mL of dichloromethane. The mixture was stirred using a magnetic stirrer (IKA, C-MAG HS4, Freiburg, Germany) for 10 min. The organic (dichloromethane) phase was separated from the aqueous phase using a separating funnel. The aqueous phase was re-extracted twice more, each with 25 mL of dichloromethane. The organic phase from each round of extraction was stored in a 250 mL beaker and evaporated in a water bath (45–50 °C) under a fume hood (Neoflow, Vol. PP1, Pathum Thani, Thailand) until the volume was slightly below 50 mL. The final volume was adjusted using a 50 mL volumetric flask. The absorbance of the organic phase was measured at 274 nm using a UV-vis spectrophotometer (Shimadzu, UV-1601, Kyoto, Japan). Dichloromethane was used as blank. Caffeine standard at different concentrations was used to prepare a standard curve (Figure S1). The measured content of caffeine is expressed in milligrams/gram dry beans.

## 3. Mathematical Model

### 3.1. Model Description and Assumptions

**Figure 1:**
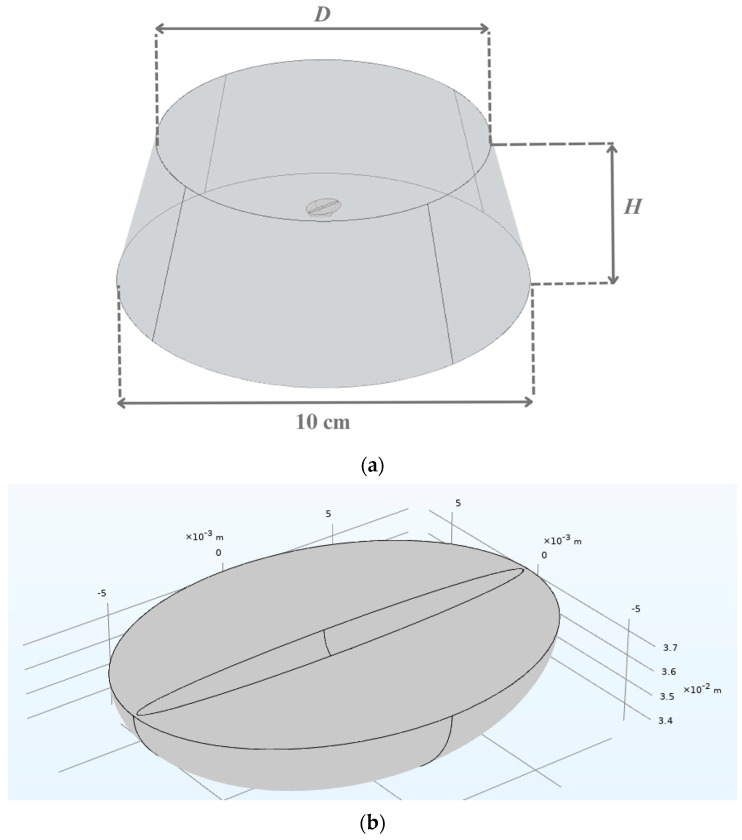
Schematic diagrams of (**a**) green coffee bean immersed in water in the flask and (**b**) green coffee bean. *H* = extraction water height in the flask, *D* = extraction water diameter in the flask.

The model geometry consisted of a green coffee bean and extraction water as the solvent. The whole content was put in a 500 mL Erlenmeyer flask, part of which is shown in Figure 1a. The measured values of the extraction water height (*H*) and diameter (*D*) in the flask at different bean-to-water ratios are given in Table 1. A green coffee bean was assumed to exhibit a semi-ellipsoidal shape with initial dimensions (height, width, and thickness) of 8.56 × 6.41 × 4.22 mm. The assumed bean geometry is shown in Figure 1b. The bean was placed at the middle of the extraction water height (at *H*/2).

To simplify the analysis, the following assumptions were made:

- The coffee bean was assumed to be an isotropic and homogeneous material.
- Initial caffeine and moisture contents as well as temperature were uniform throughout the whole bean.
- Size and volume of the bean during the extraction are a function of the moisture content. The bean was assumed to uniformly expand in all three dimensions.
- Heat transfer within the bean was negligible. The extraction process was assumed to be isothermally held at the extraction (water) temperature. Based on our preliminary experiments, the come-up time, which is the time it took the bean temperature to reach the extraction temperature, was only around 2%–4% of the total extraction time. Energy equation was therefore not required.
- Interactions among coffee beans were considered negligible. Only a single bean was therefore simulated as a representative of all the beans. Note that the difference in coffee bean sizes is not included in the model. However, the model geometry was created using the average dimensions as the representative values.

### 3.2. Governing Conservation Equations

The governing conservation equations comprise those describing mass transfer in the solid phase (coffee bean) and liquid phase (extraction water). Both caffeine and water (moisture) transport processes were modeled as the components traveled through the bean, with water acting as the solvent during the extraction. The equations for the solid phase, expressed in terms of the molar concentrations of caffeine and moisture, are given in Equations (2) and (3):

(2) $$\frac{\partial C_{c,s}}{\partial t}={\nabla \cdot (D}_{c,s}\nabla C_{c,s})$$

(3) $$\frac{\partial C_{m,s}}{\partial t}=\nabla \cdot (D_{m,s}\nabla C_{m,s})$$

where *C*_c,s_ is the molar concentration of caffeine in the solid phase (mol·m^−^^3^), *C*_m,s_ is the molar concentration of moisture in the solid phase (mol·m^−^^3^), *D*_c,s_ is the effective diffusivity of caffeine in the solid phase (m^2^·s^−^^1^), and *D*_m,s_ is the effective diffusivity of moisture in the solid phase (m^2^·s^−^^1^). For the liquid phase, the equations are similar to those of the solid phase, as given in Equations (4) and (5):

(4) $$\frac{\partial C_{c,l}}{\partial t}={\nabla \cdot (D}_{c,l}\nabla C_{c,l})$$

(5) $$\frac{\partial C_{m,l}}{\partial t}=\nabla \cdot (D_{m,l}\nabla C_{m,l})$$

where *C*_c,l_ is the molar concentration of caffeine in the liquid phase (mol·m^−^^3^), *C*_m,l_ is the molar concentration of moisture in the liquid phase (mol·m^−^^3^), *D*_c,l_ is the effective diffusivity of caffeine in the liquid phase (m^2^·s^−^^1^), and *D*_m,l_ is the effective diffusivity of moisture in the liquid phase (m^2^·s^−^^1^). The Arrhenius equation is used to describe the changes in the diffusivities of caffeine and moisture in both phases as a function of the extraction temperature:

(6) $$D_{c,s}\;or\;D_{m,s}\;or\;D_{c,l}\;or\;D_{m,l}=A\exp\left(\frac{E_{a}}{RT}\right)$$

where A is the pre-exponential factor, $E_{a}$ is the activation energy (J·mol^−^^1^), *R* is the universal gas constant (8.314 J·mol^−^^1^·K^−^^1^), and *T* is the extraction temperature (K). The values of A and $E_{a}$ for $D_{c,s}$, $D_{m,s}$, $D_{c,l}$ and $D_{c,s}$ were obtained by fitting the simulated results to the experimental data at various temperatures in the range of 40–70 °C. The results are given in Table 2.

Molar fluxes of caffeine and moisture at the interphase are described in the form of convective mass transfer fluxes, as per Equations (7) and (8):

(7) $$J_{c}=k_{c}(C_{c,i}-C_{c,l})$$

(8) $$J_{m}=k_{m}(C_{m,l}-C_{m,i})$$

where $J_{c}$ and $J_{m}$ are the molar fluxes of caffeine and moisture at the bean–extraction water interphase (mol·m^−^^2^·s^−^^1^); $C_{c,i}$ and $C_{m,i}$ are the molar concentrations of caffeine and moisture at the interphase (mol·m^−^^3^); and $k_{c}$ and $k_{m}$ are the convective mass transfer coefficients of caffeine and moisture (m·s^−^^1^). Note that the mass transfer resistance between the bean surface and bulk extraction water is incorporated into the values of $k_{c}$ and $k_{m}$, which are the typical parameters expressing the external mass transfer resistance. An Arrhenius-type equation is again used to describe the convective mass transfer coefficients of caffeine and moisture as a function of the extraction temperature and ultrasonic power:

(9) $$k_{c}\;or\;k_{m}=Aw_{c}^{-1}{\left(P+1\right)}^{0.03}exp(\frac{E_{a}}{RT})$$

where *w*_c_ is the mass fraction of caffeine in the solution (-) and P is the specific absorbed ultrasonic power (W·kg^−^^1^). The values of *A* and $E_{a}$ during CAE and UAAE were again obtained by fitting the simulated results to the experimental data. The results are also given in Table 2. Note that the value of P in the case of CAE is equal to zero. The value of 0.03 in Equation (9) was obtained by fitting the simulated results to the experimental data.

### 3.3. Volume Expansion Correlation

As mentioned earlier, the bean was assumed to uniformly expand in all three directions. The equation that is used to describe the volume expansion of the bean during both CAE and UAAE is [24]:

(10) $$\frac{V}{V_{0}}=a{\left(\frac{C_{m,s}}{C_{m,0}}\right)}^{2}+b\left(\frac{C_{m,s}}{C_{m,o}}\right)+c$$

where *V* is the volume of the bean at any instantaneous time *t* (m^3^), *V*_0_ is the initial volume of the bean (m^3^), $C_{m,o}$ is the initial molar concentration of moisture in the bean (mol·m^−^^3^), and *a*, *b*, and *c* are empirical constants. The values of the empirical constants given in Table 2 were obtained by fitting the simulated results to the experimental data. An arbitrary Lagrange–Eulerian (ALE) formulation was used to track the moving boundaries.

### 3.4. Initial and Boundary Conditions

The initial and boundary conditions that were used to solve Equations (2)–(5) are as follows.

Initial conditions (at *t* = 0):

- $C_{c,s}$,$C_{m,s}$ = initial concentrations of caffeine and moisture in the solid phase, respectively.
- $C_{c,l}$,$C_{m,l}$ = initial concentrations of caffeine and moisture in the liquid phase, respectively.

When the extraction water was changed, the replacement interval varied between 1 and 4 h. After each change, the caffeine and moisture concentrations in the solid phase were reset to the volume-average concentrations from the previous extraction cycle, while the caffeine and moisture concentrations in the liquid phase were set back to their initial values.

Boundary conditions:

- $J_{c}$, $J_{m}$ are as listed in Equations (7) and (8).
- $\nabla C_{c}$, $\nabla C_{m}$ = 0 at the bean center.

### 3.5. Model Implementation

**Figure 2:**
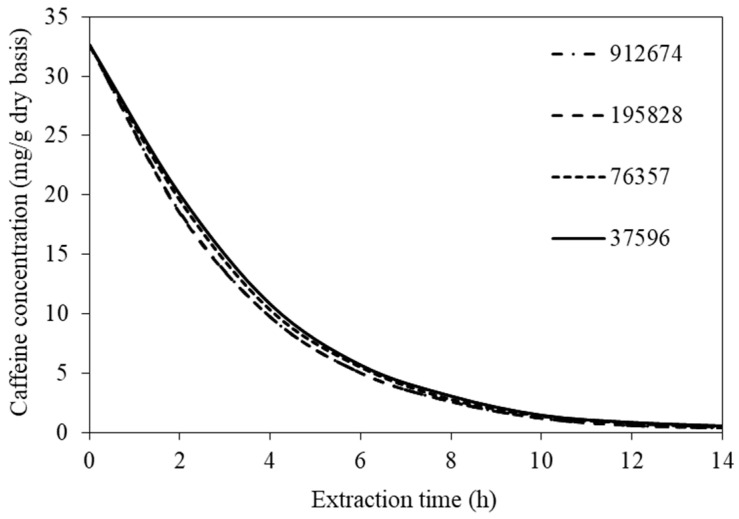
Evolutions of caffeine concentration at different numbers of mesh elements.

COMSOL Multiphysics^TM^ version 6.0 (Comsol AB, Stockholm, Sweden) was used to solve all the model equations. To obtain mesh-independent solutions, the various mesh densities were tested. The predicted volume-average caffeine concentrations were compared. The values at the highest extraction temperature (70 °C) were compared since they represented the values obtained when the steepest gradients of concentrations existed. Maximum differences in the values of the caffeine concentration were less than 2% when 195,828 and 912,674 mesh elements were used (see Figure 2). Therefore, 195,828 mesh elements were selected. The simulation time requirements were 30–60 min on a MacBook Pro with Intel**^®^** Core™ i9 (2.3 GHz), 8-core processors, and 32 GB RAM.

## 4. Results and Discussion

### 4.1. Model Validation

**Figure 3:**
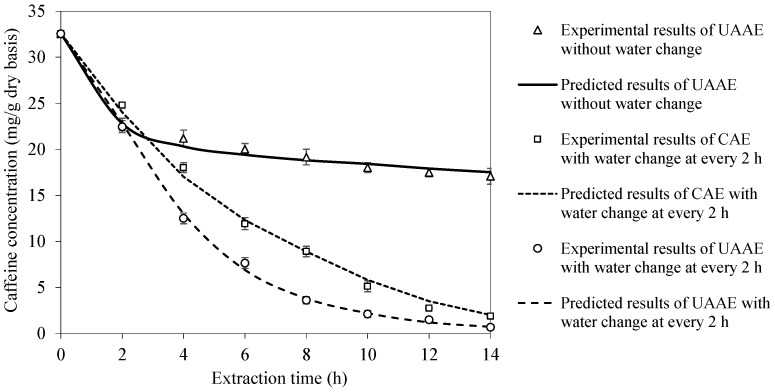
Model validation in terms of caffeine concentration evolution at extraction temperature of 70 °C and bean-to-water ratio of 1:3.

Experimental UAAE data at the extraction temperature of 70 °C and bean-to-water ratio of 1:3 were used to validate the proposed mathematical model in terms of the caffeine concentration evolution. Such experimental conditions were chosen because they provided the highest caffeine removal rate and at the same time resulted in the least extensive deterioration in bean structure. The ability of the model to capture the effect of extraction water change was also verified for CAE and UAAE. The model is seen to be capable of well predicting the experimental data. The maximum differences between the experimental and predicted results are less than 5% in all cases, as shown in Figure 3.

### 4.2. Effect of Extraction Temperature on Caffeine Concentration Evolution

To numerically investigate the effect of extraction temperature on the caffeine concentration evolution, the temperature was varied in the range of 50–80 °C while maintaining the bean-to-water ratio at 1:3. Although higher extraction temperatures (higher than 80 °C) would have increased the amount of the extractable caffeine, our preliminary experimental data revealed that a temperature higher than 80 °C resulted in an excessively high rate of water evaporation. This caused inconvenience in performing the extraction. The beans were also noted to germinate and exhibited significant changes in terms of color (getting paler) and texture (getting softer) at the higher temperatures.

**Figure 4:**
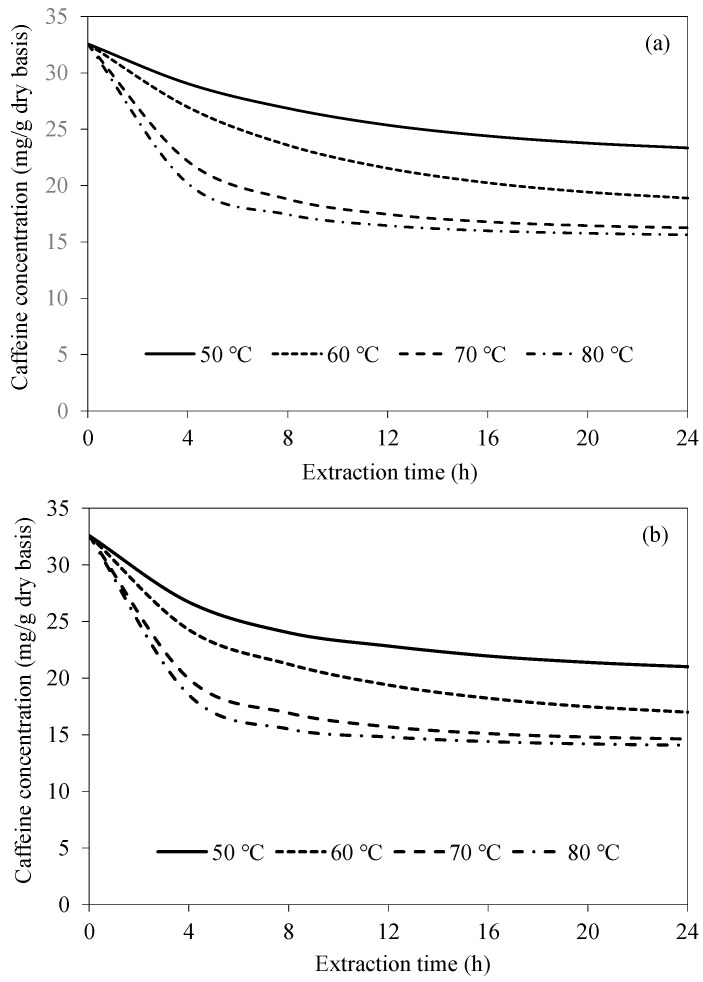
Evolutions of caffeine concentration at different extraction temperatures at a fixed bean-to-water ratio of 1:3 for (**a**) CAE and (**b**) UAAE.

Figure 4 shows the simulated caffeine concentration evolutions at various extraction temperatures for CAE and UAAE. Caffeine concentration more rapidly decreases during an early period of extraction (within 4 h) due to the higher caffeine concentration gradient between the bean surface and the extraction water. As the extraction process proceeds, caffeine concentration more slowly decreases due to the lower concentration gradient. Caffeine would also need to travel farther from the interior of the bean to the surface as the extraction process proceeds. At a higher extraction temperature, the rate of caffeine removal is higher. This is because the solubility of caffeine in water increases with temperature from around 6.97 g/100 mL at 50 °C to 76.6 g/100 mL at 80 °C [25,26]. Diffusivity of caffeine within the bean is also higher at a higher temperature, resulting in a more rapid transport of caffeine from within the bean to the surface. However, when the temperature increases from 70 °C to 80 °C, the caffeine concentrations within the bean after 12 h extraction for both CAE and UAAE remain unchanged. This is probably because the equilibrium between the concentration of caffeine in the bean and that in the extraction water has already been reached. A previous study also reported that beans were more damaged at an extraction temperature of 80 °C than at 70 °C. Thus, the extraction temperature of 70 °C is the most appropriate choice for either CAE or UAAE [18].

When comparing CAE and UAAE at any extraction temperature, the rate of caffeine concentration reduction with the latter is always higher. This is because of acoustic cavitation produced by ultrasonic waves. Such a cavitation in turn results in the creation and implosion of microbubbles, which produces shock waves and microjets that cause bean surface disruption (and probably cellular disruption as well). Caffeine can therefore more effectively travel from the bean to the extraction water. Prior to microbubble implosion, the pulsating actions of bubble growth through sound wave cycles may also cause acoustic streaming, which promotes agitation of the extraction water. This leads to reduced external mass transfer resistance [27]. The efficiency of caffeine removal in the case of UAAE is consistently around 10% higher than that in the case of CAE at every extraction temperature and time.

### 4.3. Effect of Bean-to-Water Ratio on Caffeine Concentration Evolution

Based on the abovementioned results, an extraction temperature of 70 °C was selected to numerically investigate how the caffeine concentration within the bean evolves at different bean-to-water ratios. The ratio was varied in the range of 1:1 to 1:4.

**Figure 5:**
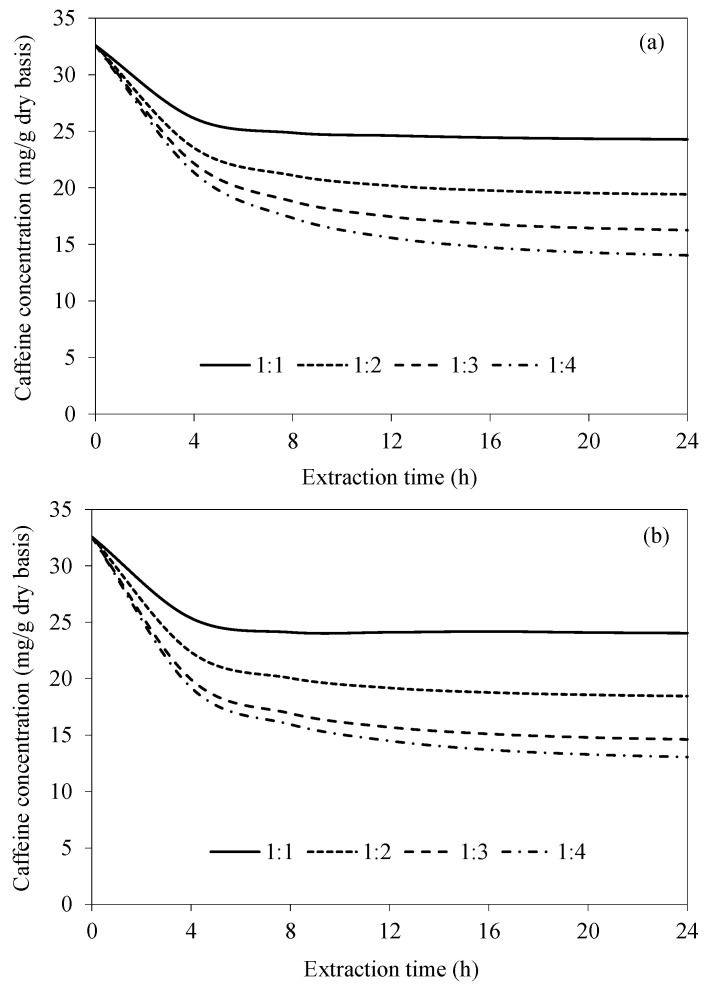
Evolutions of caffeine concentration at different bean-to-water ratios at a fixed temperature of 70 °C for (**a**) CAE and (**b**) UAAE.

Figure 5 shows the simulated caffeine concentration evolutions at various bean-to-water ratios for CAE and UAAE. In all cases, the caffeine concentration rapidly decreases during the first 4 h and then more slowly decreases afterwards. An increase in the bean-to-water ratio results in a much more rapid reduction in caffeine. This is simply because of the higher caffeine concentration gradient between the bean surface and the extraction water at the higher bean-to-water ratios. When the extraction time is longer than 16 h, the caffeine concentration is noted to remain unchanged, especially in the case of CAE. Again, this is probably because the equilibrium between the caffeine concentration in the bean and that in the extraction water has been reached. As the bean-to-water ratio increases from 1:3 to 1:4, the maximum difference in the intra-bean caffeine concentration is only around 5%, as can be seen in Figure 5b. This indicates that increasing the water mass from three times to four times the coffee bean mass does not cause a significant difference in the caffeine removal rate. This confirms our preliminary experimental finding that the most appropriate ratio for maximizing the caffeine removal is 1:3.

When comparing the efficiency of the two extraction methods, the remaining caffeine concentration within the bean in the case of UAAE was always lower than that in the case of CAE. This is due to the effect of acoustic cavitation, as mentioned earlier. The average differences in the residual caffeine concentrations within the beans extracted by CAE and UAAE at bean-to-water ratios of 1:1, 1:2, 1:3 and 1:4 are 2%, 5%, 10% and 7%, respectively. A decrease in the percentage difference at a bean-to-water ratio of 1:4 is probably due to the lower specific ultrasonic intensity (in W/g) in an excessive volume of water [28].

### 4.4. Effect of Extraction Water Change Frequency on Caffeine Concentration Evolution

As can be seen from the abovementioned simulation results, the efficiency of caffeine removal, either for CAE or UAAE, without water change at any temperature and bean-to-water ratios is low. This is because the equilibrium between the caffeine concentration in the bean and that in the extraction water has been reached too early. To maintain a higher caffeine concentration gradient between the bean surface and the bulk of the extraction water, the idea of changing the extraction water at time intervals was tested. The frequency of extraction water change was varied in the range of 1–4 h. A fixed extraction temperature of 70 °C and bean-to-water ratio of 1:3 were used in the simulation. These two latter choices were based on the results of the earlier simulations.

**Figure 6:**
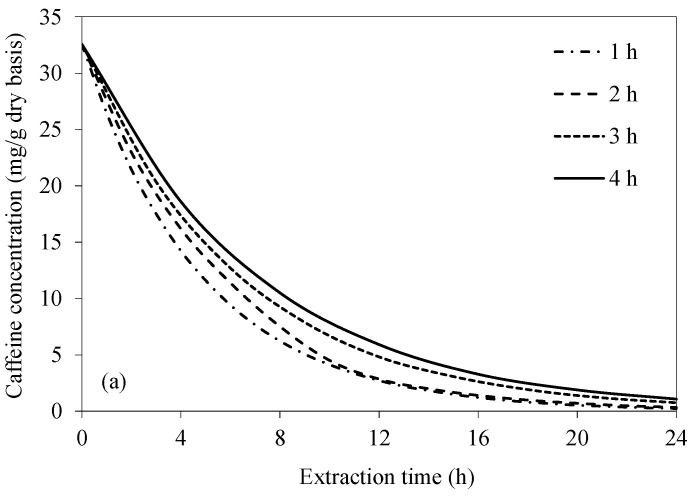

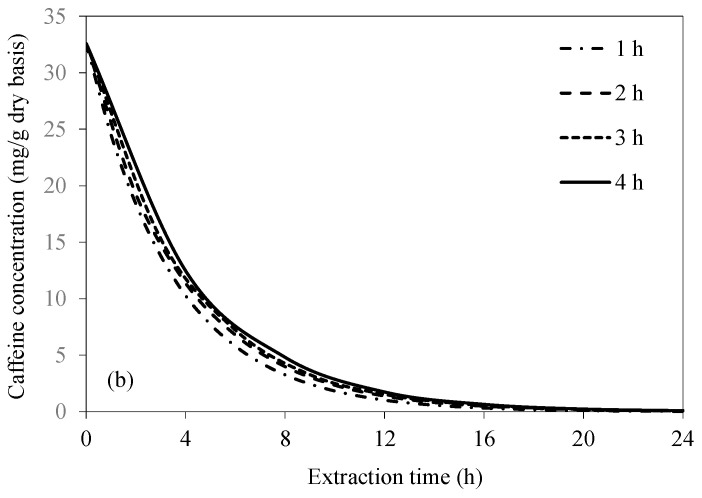
Evolutions of caffeine concentration at different water change frequencies at a fixed temperature of 70 °C and bean-to-water ratio of 1:3 for (**a**) CAE and (**b**) UAAE.

Figure 6 shows the simulated caffeine concentration evolutions at various frequencies of extraction water change. Caffeine concentration within the bean significantly decreases with an increase in the frequency of extraction water change, especially in the case of UAAE. This is because the caffeine concentration gradient between the bean surface and the bulk of the extraction water remains higher during the extraction process. The results are more evident in the case of UAAE, since the intensity of acoustic cavitation remains constantly high when the extraction water is replaced with fresh water at different time intervals. This is ascribed to the fact that more soluble solids, i.e., the extracted caffeine and other constituents, in the extraction water act to reduce the intensity of acoustic cavitation [29]. The results indicate that the condition that yields almost complete caffeine removal (99.9%) as per the European Union standard is UAAE for 24 h, with extraction water change at every 1 h interval. Note that an increase in the caffeine removal from 99.0% to 99.9% requires 33% of the total UAAE time. Complete caffeine removal does not occur in the case of CAE. The highest caffeine removal is around 99.3% after 24 h of the extraction, even with extraction water change at every 1 h interval.

If the criterion set forth by the USDA that requires at least 97.5% caffeine removal is to be met, the required extraction time in the case of UAAE would be 13, 14, 15, and 16 h when the extraction water is changed at every 1, 2, 3, and 4 h, respectively. The reason for finally choosing to change the extraction water at 1 h intervals is simply that doing so would result in the shortest overall extraction duration. Note that the equilibrium between the caffeine concentrations at the bean surface and in the bulk extraction water would be reached at a time between 1 and 2 h after the start of the extraction process. A 1 h interval was therefore chosen to speed up the process.

The caffeine concentration within the bean is lower for UAAE than CAE at any tested frequency of extraction water change. Such an observation is especially more obvious during the first 12 h. After 12 h, UAAE tends to have reached the equilibrium, while CAE could still remove caffeine at a higher rate, leading to a smaller difference in the residual caffeine concentration of the beans extracted by CAE and UAAE at the same extraction time.

Based on the simulation results, the most appropriate condition that provides the target caffeine removal as per the European Union standard (requiring 99.9% caffeine removal) is UAAE with water change at every 1 h interval at 70 °C. The bean-to-water ratio should be 1:3 and the extraction should be conducted for 24 h. This condition is also suggested if the US standard (requiring 97.5% caffeine removal) is to be met, for which the required extraction time would be only 13 h. Note that the latter condition was experimentally verified. The results show that 97.72 ± 0.07% of caffeine within the beans could be removed, satisfying the requirement of the US standard. Due to the limitation related to our laboratory regulation, we were not be able to experimentally verify if the 24 h extraction time would result in the target caffeine removal as per the European Union standard.

## 5. Conclusions

A mathematical model capable of predicting caffeine concentration evolution during CAE and UAAE with or without intermittent extraction water change is proposed. After successful validation with the experimental data, the model was used to predict the caffeine concentration evolution within a whole green robusta coffee bean during both CAE and UAAE. The effects of temperature, bean-to-water ratio, and frequency of extraction water change on the evolution of caffeine concentration were investigated to arrive at the most appropriate condition that provides the target caffeine removal as per the European Union and US standards of decaffeinated coffee.

The extraction efficiency of UAAE is 10% higher than that of CAE in all cases. The required UAAE time to meet the caffeine removal levels as per the US and European Union standards is 28%–38% shorter than that required by CAE. An extraction temperature of 70 °C, bean-to-water ratio of 1:3, and extraction water change frequency of 1 h are suggested as the most appropriate conditions for UAAE. The predicted required extraction time of UAAE at such a condition is 13 h to meet the US standard, which requires 97.5% caffeine removal, and 24 h to meet the European Union standard, which requires 99.9% caffeine removal. The suggested conditions to meet the US standard were experimentally verified and found to yield similar results to those obtained via the simulation.

Note that the present study focused only on a single bean, which clearly does not reflect actual interactions between beans in a real system. Further study should therefore develop a model that takes into account interactions between beans and verify the model with a large-scale extraction process. Assessment of impacts of the extraction process on sensory characteristics of the beans as well as investigation of the process applicability to other coffee varieties should also be conducted.

## Figures and Tables

**Table 1:** Measured values of extraction water height (*H*) and diameter (*D*) in the flask at different bean-to-water ratios.

Bean-to-Water Ratio	*H* (cm)	*D* (cm)
1:1	9.5	3.5
1:2	8.5	5.5
1:3	7.5	7.5
1:4	5.5	11.5

**Table 2:** Values of Arrhenius parameters and empirical constants.

Parameter	Value	
	*A*	*E*_a_ (J/mol)
$D_{c,b}$	3.6 × 10^−9^	12,000
$D_{m,b}$	7.2 × 10^−9^	12,000
$D_{c,l}$	5.1 × 10^−9^	11,000
$D_{m,l}$	3.4 × 10^−8^	8000
$k_{c}$	6.6 × 10^−5^	8000
$k_{m}$	3.8 × 10^−5^	6000
Value		
*a*	*b*	*C*
0.30	0.14	0.60

## Data Availability

The original contributions presented in this study are included in the article/Supplementary Materials. Further inquiries can be directed to the corresponding author.

## Supplementary Materials

The following supporting information can be downloaded at https://www.mdpi.com/article/10.3390/foods14111956/s1. Figure S1. Calibration curve for caffeine content determination at 274 nm.
