# Supplementary material for: Numerical Investigation of Conventional and Ultrasound-Assisted Aqueous Extraction of Caffeine from Whole Green Robusta Coffee Beans: Extraction Enhancement via Changing of Extraction Water
Source: Foods. 2025 May 30;14(11):1956. doi: 10.3390/foods14111956 (PMC12155289; doi:10.3390/foods14111956)
Supplement: Supplementary file 1 [file foods-14-01956-s001.zip › foods-3585056-supplementary.pdf]

Supplementary materials

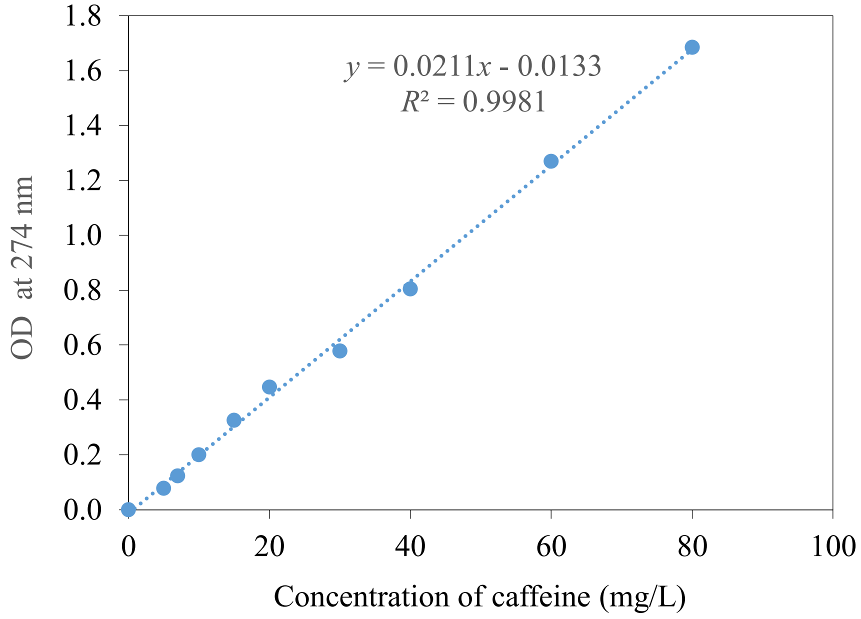

Figure S1. Calibration curve for caffeine content determination at 274 nm.
